# Patients with combined pelvic and spinal injuries have worse clinical and operative outcomes than patients with isolated pelvic injuries analysis of the German Pelvic Registry

**DOI:** 10.1186/s12891-022-05193-0

**Published:** 2022-03-15

**Authors:** Luis Navas, Natalie Mengis, Alexander Zimmerer, Jules-Nikolaus Rippke, Sebastian Schmidt, Alexander Brunner, Moritz Wagner, Andreas Höch, Tina Histing, Steven C. Herath, Markus A. Küper, Benjamin Ulmar

**Affiliations:** 1 ARCUS Sportklinik, Rastatterstraße 17–19, 72175 Pforzheim, Germany; 2 Orthopädische Klinik PaulinenhilfeDiakonieklinikum Stuttgart, Rosenbergstraße 38, 70176 Stuttgart, Deutschland; 3 Department of Orthopedics and Orthopedic Surgery, University Medicine Greifswald, Ferdinand-Sauerbruch-Straße, 17475 Greifswald, Germany; 4 Bezirkskrankenhaus St. Johann in Tirol, Bahnhofstrasse 14, 6380 St. Johann, Tirol Austria; 5 Department of Orthopedics, Trauma and Plastic Surgery, University of Leipzig, Liebigstraße 20, 04103 Leipzig, Germany; 6 Department for Traumatology and Reconstructive Surgery, BG Trauma Center, University of Tübingen, Schnarrenbergstraße 95, 72,076 Tübingen, Germany

**Keywords:** Pelvic trauma, Pelvic ring fracture, Acetabular fracture, Spine injury, Postoperative reduction, Mata grading

## Abstract

**Background:**

Pelvic fractures are often associated with spine injury in polytrauma patients. This study aimed to determine whether concomitant spine injury influence the surgical outcome of pelvic fracture.

**Methods:**

We performed a retrospective analysis of data of patients registered in the German Pelvic Registry between January 2003 and December 2017. Clinical characteristics, surgical parameters, and outcomes were compared between patients with isolated pelvic fracture (group A) and patients with pelvic fracture plus spine injury (group B). We also compared apart patients with isolated acetabular fracture (group C) versus patients with acetabular fracture plus spine injury (group D).

**Results:**

Surgery for pelvic fracture was significantly more common in group B than in group A (38.3% vs. 36.6%; *p* = 0.0002), as also emergency pelvic stabilizations (9.5% vs. 6.7%; *p* < 0.0001). The mean time to emergency stabilization was longer in group B (137 ± 106 min vs. 113 ± 97 min; *p* < 0.0001), as well as the mean time until definitive stabilization of the pelvic fracture (7.3 ± 4 days vs. 5.4 ± 8.0 days; *p* = 0.147). The mean duration of treatment and the morbidity and mortality rates were all significantly higher in group B (*p* < 0.0001). Operation time was significantly shorter in group C than in group D (176 ± 81 min vs. 203 ± 119 min, *p* < 0.0001). Intraoperative blood loss was not significantly different between the two groups with acetabular injuries. Although preoperative acetabular fracture dislocation was slightly less common in group D, postoperative fracture dislocation was slightly more common. The distribution of Matta grades was significantly different between the two groups. Patients with isolated acetabular injuries were significantly less likely to have neurological deficit at discharge (94.5%; *p* < 0.0001). In-hospital complications were more common in patients with combined spine plus pelvic injuries (groups B and D) than in patients with isolated pelvic and acetabular injury (groups A and C).

**Conclusions:**

Delaying definitive surgical treatment of pelvic fractures due to spinal cord injury appears to have a negative impact on the outcome of pelvic fractures, especially on the quality of reduction of acetabular fractures.

## Introduction

In the case of a polytrauma patient, all possibly life-threatening injuries must be identified and treated as quickly as possible. Spinal cord injury should always be considered in patients with multiple injuries, even if no neurological deficit is present [1]. About 5% of polytrauma patients with pelvic and/or acetabular fracture will have spinal injury [29, 42]. Pelvic fractures as well as spine injuries occur generally in road traffic accidents or falls from heights, and the combination of both injuries can be life-threatening due to the force exerted to cause these injuries [18, 54]. Approximately 55% of spinal cord injuries occur in the cervical region, 15% in the thoracic region, 15% in the thoracolumbar junction, and 15% in the lumbosacral region. Up to 10% of patients with a cervical spine fracture will have a second, non-contiguous spine fracture [1, 13].

Due to the complex anatomy of the pelvis, pelvic fractures are often divided into pelvic ring fractures and acetabular fractures, as depending on the area of injury the surrounding tissues play an important role in concomitant injuries. Due to these characteristics and how scarcity these are, the treatment of such fractures depends on a certain degree of surgical expertise.

The most favorable time for osteosynthetic stabilization—performed to restore the pelvic ring stability and/or anatomic acetabular restoration—is determined by the gravity of adjuvant injuries and hemodynamic stability.

The stability of the pelvic ring is graded using the Tile classification, for which the integrity of the posterior pelvic ring dictates the stability grade of the entire pelvic ring [34]. Tile A lesions can be managed conservatively, but Tile B or C lesions will require surgical stabilization, usually by percutaneous insertion of sacroiliac screws for the posterior pelvic ring [10, 21]. In some cases, open reduction and internal fixation (ORIF) for the anterior pelvic ring may be required; however, an important requisite for ORIF is a hemodynamically stable patient. A supraacetabular external fixator is sometimes used for temporary or definitive treatment of the anterior pelvic ring injuries [43, 44].

In acetabular fractures, the essential objective of treatment is anatomical restoration of the joint line to avoid development of post-traumatic osteoarthritis.

﻿ORIF is the gold standard treatment for displaced acetabular fractures. The quality of reduction achieved is recorded by the Matta grading score, which describes the postoperative step-off in the joint in Grade 1 (anatomical reconstruction: < 2 mm), Grade 2 (imperfect reduction: 2 – 3 mm), and Grade 3 (poor reduction > 3 mm) [3, 19, 28, 37, 48].

In patients with combined pelvic fracture and spine injury, treatment of the pelvic fracture is often delayed until the life-threatening spine injury has been dealt with, but whether this results in worse postoperative outcome of the pelvic fracture is not known [33, 41, 42]. The aim of this multicenter cohort study was to investigate how the postoperative outcome, follow-up and surgical treatment are influenced with a concomitant spine injury.

Our primary hypothesis was that an associated spine injury leads to delayed definitive surgical treatment of the pelvic fracture and that this unfavorably affects reduction quality of acetabular fractures.

## Patients and methods

The Working Group Pelvis (AG Becken) of the German Society for Traumatology (Deutsche Gesellschaft für Unfallchirurgie) established a multicenter prospective registry in 1991 to improve the quality of care for pelvic and acetabular injuries. The Pelvic III Working Group prospectively records all pelvic and acetabular fractures in the participating hospitals (currently, 39 hospitals) [7, 8, 26]. ﻿The database is maintained since 2018 by the AUC GmbH (Academy of Trauma Surgery [Akademie der Unfallchirurgie]) with the sponsoring of the German Society for Traumatology and the Data used were collected in Bern, Switzerland by MEMdoc (medical Registries and Data Linkage). The German Pelvic Registry (GPR) has been approved by the Ethics Committee of the Chamber of Physicians of the Federal State of Saarland (No. 29/14). Inclusion criteria for the registry are pelvic ring and/or acetabular fracture and informed consent. Follow-up is determined for each patient individually conform to the duration of stationary treatment for the pelvic injury.﻿ The data of 16.359 patients with pelvic fractures were recorded correctly and entirely in the GPR from January 2003 to December 2017. These included 4.547 (27.8%) with acetabular fracture. We identified those with and without associated spine injury. In the GPR, spine injury is defined based on the criteria of the Injury Severity Scores (ISS) as an Abbreviated Injury Score (AIS Spine) > 0 for each spine segment (cervical, thoracic and lumbar) [22]. The data of the patients were extracted for retrospective analysis.

The Ethics Committee of the Eberhard-Karls-University in Tübingen, Germany, approved this cohort study (No. 968/2018BO2).

### Evaluated parameters

The following parameters were analyzed:

- Age
- Gender
- Injury Severity Score (ISS)
- Hemoglobin level (Hb) at admission
- Systolic blood pressure (SBP) at admission
- Number of emergency stabilizations
- Number of definitive surgical stabilizations
- Time until emergency fracture stabilization (in minutes)
- Time until definitive stabilization (in days)
- Length of hospital stay (in days)
- Overall complication rate (except osteosynthesis-associated complications)
- Rate of osteosynthesis-associated complications
- Mortality

The following complications were evaluated:

- Hemorrhagic events
- Thromboembolic events
- Surgical site infection (superficial and deep)
- Fracture-associated neurologic complications (preoperatively existing)
- Iatrogenic neurologic complication
- Pulmonary complications
- Cardiac complications
- Multiorgan failure

The following complications associated with osteosynthesis were evaluated:

- Implant loosening
- Implant failure
- Secondary displacement of the fracture after definitive surgical fixation

To evaluate the quality of surgery in the acetabular fractures subgroup, the following surgical parameters were evaluated, focusing on the quality of the postoperative reposition:

- Lenght of surgery (minutes)
- Blood loss (milliliters)
- Preoperative maximal fracture step-off (millimeters)
- Postoperative maximal fracture step-off (millimeters)
- Reduction quality based on Matta classification

Pre- and postoperative fracture step-offs were measured on pelvic radiographs (including oblique/iliac views) or CT scans by surgeons experienced in acetabular injuries. The maximum step-offs were recorded into the database.

### Statistical analysis

Normally distributed data were expressed as means ± standard deviation, and non-normally distributed data as medians (range). Differences between group median values were analyzed using ANOVA with the Bonferroni and Tukey correction, as well as t-test and chi-square test. A *p* value < 0.05 was accepted as statistically significant. Data collection and tabulation were performed using Microsoft Excel.

Statistical analysis was performed using SPSS Statistics® (SPSS GmbH, Munich), with the help of Prof. Dr. Wolf-Dieter Heller (KIT Karlsruhe Institute of Technology).

## Results

**Fig. 1:**
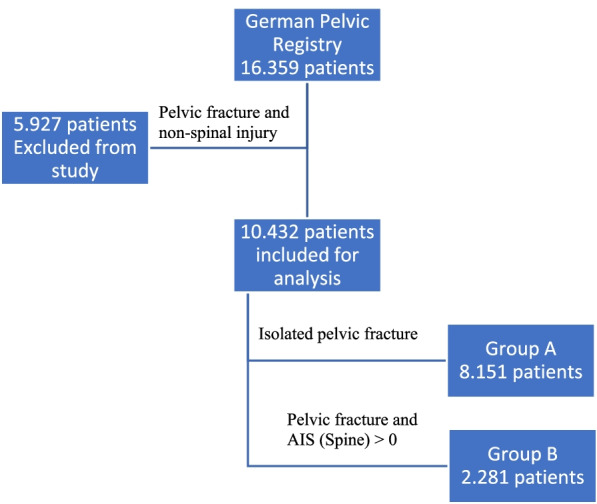
Patient’s cohort from the GPR. Group A include patients with isolated pelvic fracture. Group B include patients with pelvic fracture plus spine injury. The remaining 5.927 patients were excluded from the study. Spine injury was defined as AIS (spine) > 0

Of the 16.359 patients with pelvic fracture, 5. 927 (36.2%) had associated non-spinal injuries and so were excluded. Among the remaining patients, 8. 151 (49.8%, group A) had isolated pelvic ring and/or acetabular fracture and 2. 281 (13.6%, group B) had pelvic ring and/or acetabular fracture plus spine injury (Fig. 1).

**Fig. 2:**
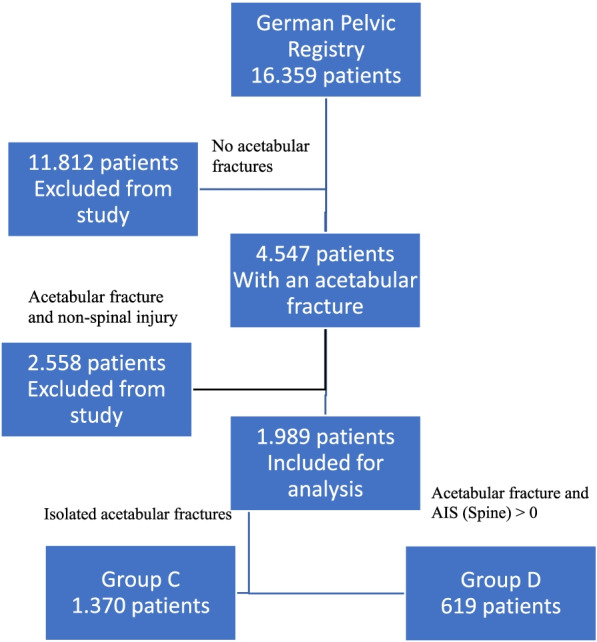
Patient’s cohort from the GPR. Group C include patients with isolated acetabular fracture. Group D include patients with combined acetabular fracture and spine injury. The remaining 11.812 patients were excluded from the study. Spine injury was defined as an AIS (Spine) > 0

Of the 4. 547 patients with acetabular fractures, 2.558 (56.3%) had associated non-spinal injuries and so were excluded from the analysis. While 1.370 (30.1%; group C) had isolated acetabular fracture, 619 (13.6%; group D) had acetabular fracture plus spine injury (Fig. 2).

### Basic data and fracture distribution

Mean age was significantly lower and ISS significantly higher in group B than in group A (*p* < 0.0001 for both). Pelvic ring fractures were the most common fracture type, being present in 73% of the total cohort. The dispersion of fracture types (isolated pelvic ring fracture, isolated acetabular fracture, or combined pelvic ring and acetabular fracture) was comparable in the two groups. Nevertheless, the proportion of unstable pelvic ring fractures (Tile B or C) was significantly lower in group A than in group B (55% vs. 92%, *p* < 0.0001; Table 1).

**Table 1:** Comparison of the fracture distribution and demographic data between patients with isolated pelvic fracture (group A) and combined spine/pelvic injury (group B). Values are shown as n (%) or as the mean ± standard deviation [range]; the data of ISS (Injury Severity Score) are the median values

	**GROUP A (*n* = 8 151**	**GROUP B (*n* = 2 281)**	***p***
AGE, YEARS, MEAN ± SD [RANGE]	70.5 ± 20.4 [4–105]	49.8 ± 21.3 [6–102]	< 0.0001
SEX, % (n)			< 0.0001
MALE	35.5% (2.893)	58.1% (1.325)	
FEMALE	65.5% (5.258)	39.4% (899)	
ISS	9	28	< 0.0001
TYPE OF PELVIC fracture, % (n)			0.31
PELVIC RING FRACTURE	73.1% (5.956)	54.9% (1.252)	
ACETABULAR FRACTURE	23.3% (1.898)	37.6% (858)	
COMBINED PELVIC RING + ACETABULAR FRACTURE	3.6% (297)	5.0% (114)	
TYPE OF PELVIC RING FRACTURE, % (n)			< 0.0001
STABLE (TILE A)	44.8% (2.669)	7.8% (178)	
UNSTABLE (TILE B/C)	55.2% (3.287)	89.7% (2.046)	

Concerning to the hemodynamic status of patients at the hospital admission, only data for 49 patients in group A and for 353 patients in group B were available and valid. Hemodynamic instability (Hb < 8.0 g/dL and/or SBP < 100 mm Hg) was significantly more common in group B patients than in group A patients (56% vs. 15%, *p* < 0.0001). Surgery for pelvic injury was performed more often in group B than in group A (38.3% vs. 36.6%; *p* = 0.0002), as also emergency pelvic stabilizations (9.5% vs. 6.7%; *p* = 0.176). The mean time until emergency stabilization was longer in group B (137 ± 106 min vs. 113 ± 97 min; *p* < 0.0001), as well as the mean time until definitive stabilization of the pelvic fracture (7.3 ± 4 days vs. 5.4 ± 8.0 days; p = 0.147). The mean duration of treatment and the morbidity and mortality were all significantly higher in group B than in group A (*p* < 0.0001; Table 2).

**Table 2:** Association of the clinical course between patients with isolated pelvic fracture (group A) and patients with combined spine/pelvic injury (group B). Values are shown as n (%), as a mean (μ) or as the mean ± standard deviation (range)

	Group A (*n* = 8.151)	Group B (*n* = 2.281)	*p*
Hemodynamical status at admission			
Hb < 8.0 g/dL	12.2% (6/49)	37.9% (139/367)	< 0.0001
SBP < 100 mm Hg	17.1% (7/41)	58.3% (214/367)	< 0.0001
Operative pelvic stabilization			
Emergency stabilization	6.7% (547)	9.5% (217)	0.176
Definitive pelvic fixation	29.9% (2 440)	28.8% (656)	0.251
Time till emergency stabilization (min)	113 ± 97 (2–420)	137 ± 106 (3–540)	< 0.0001
Time till definitive fixation (days)	5.4 ± 8.0 (0–42)	7.3 ± 4 (0–32)	0.147
Clinical course			
Length of hospital stay (days)	13 ± 14 (0–213)	28.8 ± 25.6 (0–256)	< 0.0001
Overall morbidity	9.9% (805)	19.0% (434)	< 0.0001
Overall mortality	1.9% (157)	7.7% (176)	0.0001
Blood units within 6 h (μ)	3.6	5.9	< 0.0001
Blood units between 7–12 h (μ)	1.1	2.4	< 0.0001
Blood units between 13–24 h (μ)	1.15	1.8	0.532

### Quality of surgery in acetabular fractures

﻿Operation time was significantly shorter in group C than in group D (176 ± 81 min vs. 203 ± 119 min, *p* < 0.0001), but intraoperative blood loss was not significantly different between the two groups. Although preoperative fracture dislocation was slightly less common in group D, postoperative fracture dislocation was slightly more common. The distribution of Matta grades was significantly different between the two groups (Table 3).

**Table 3:** Association of clinical and surgical outcomes between patients with isolated acetabular fractures (group C) and patients with combined acetabular fracture and spinal injury. Data are presented as means ± standard deviation (range) or % (n)

	Group C (*n* = 1.370)	Group D (*n* = 619)	p
Duration of surgery (min)	176 ± 81 (60–760)	203 ± 119 (4–769)	< 0.0001
Blood loss (mL)	600 ± 511 (100–3.000)	627 ± 562 (100–3.000)	0.532
Step-off preoperatively (mm)	7.6 ± 8.1 (0–160)	6.7 ± 8.5 (0–50)	0.05
Step-off postoperatively (mm)	1.2 ± 2.5 (0–33)	2 ± 2.8 (0–25)	0.32
Quality of reduction by Matta score			
Grade 1: 0–2 mm residual step (anatomical)	84.0% (982)	50% (178)	< 0.0001
Grade 2: 2–3 mm residual step (imperfect)	4.9% (57)	24.5% (87)	< 0.0001
Grade 3: > 3 mm residual step (poor)	8.5% (100)	16.9% (60)	< 0.0001
No postoperative data available	2.6% (30)	8.6% (30)	

### Spinal injury

Of the 2.224 patients in group B, 380 (17%) had isolated cervical spine injury, 680 (31%) had isolated thoracic spine injury, and 1.224 (52%) had isolated lumbar spine injury. While 84 patients had combined cervical and thoracic spine injuries, 103 had combined cervical and lumbar spine injuries, and 340 had combined thoracic and lumbar spine injuries. A total of 479 patients had injury of all three spinal segments (cervical, thoracic, and lumbar).

### Neurological deficits and other complications

Respecting the neurological disability of the patients at admission within the groups, the patients with isolated acetabular injuries present a significantly lower neurological disability at discharge (94.5%; *p* < 0.0001). Compared to the other groups, it was a significantly higher neurological disability at discharge (group B: 843 vs. 862 patients; group C: 74 vs. 99 patients; group D: 270 vs. 280 patients; *p* < 0.0001). Complications were more common in patients with spine plus pelvic injuries (groups B and D) than in patients with isolated pelvic injury (groups A and C) (Table 4).

**Table 4:** Neurological disability and complications in patients with isolated pelvic injuries (group A), patients with a combined spine/pelvic injury (group B), patients with isolated acetabular fractures (group C), and patients with combined spine/acetabular injury (group D). Values are % (n)

	Group A (*n* = 8.151)	Group B (*n* = 2.281)	Group C (*n* = 1.370)	Group D (*n* = 619)
Neurological deficit at admission				
None	95.95% (7.821)	73.98% (1.638)	95.91% (1.314)	70.60% (437)
Unknown	0.48% (39)	19.15% (424)	2.26% (31)	21.32% (132)
L1	0	1.13% (25)	0	1.62% (10)
L2	0.04% (3)	0.90% (20)	0	1.13% (7)
L3	0.01% (1)	0.99% (22)	0.15% (2)	1.29% (8)
L4	0.02% (2)	1.67% (37)	0.29% (4)	2.42% (15)
L5	0.02% (2)	3.25% (72)	0.66% (9)	4.20% (26)
S1	0.06% (5)	3.12% (69)	0.36% (5)	3.72% (23)
S2	0.04% (3)	1.63% (36)	0.29% (4)	1.94% (12)
S3–5	0.02% (2)	1.13% (25)	0.15% (2)	1.45% (9)
Bladder sphincter	0.05% (4)	1.72% (38)	0.07% (1)	0.81% (5)
Rectum sphincter	0.01% (1)	1.36% (30)	0.07% (1)	0.81% (5)
Peripheral nerves	0.04% (3)	2.03% (45)	1.09% (15)	2.91% (18)
Neurological disability at discharge				
None	92.41% (7.532)	79.49% (1.760)	94.45% (1.294)	76.58% (474)
Unknown	0.23% (19)	9.58% (212)	1.17% (16)	9.37% (58)
L1	0	1.49% (33)	0.07% (1)	2.26% (14)
L2	0.04% (3)	1.45% (32)	0.22% (3)	1.94% (12)
L3	0.02% (2)	1.90% (42)	0.44% (6)	3.23% (20)
L4	0.04% (3)	2.71% (60)	0.44% (6)	3.39% (21)
L5	0.05% (4)	4.88% (108)	1.46% (2)0	6.30% (39)
S1	0.06% (5)	4.47% (99)	0.58% (8)	5.17% (32)
S2	0.04% (3)	2.48% (55)	0.29% (4)	2.91% (18)
S3 – 5	0.02% (2)	1.81% (40)	0.15% (2)	2.26% (14)
Bladder sphincter	0.01% (1)	2.39% (53)	0	1.62% (10)
Rectum sphincter	0.01% (1)	2.03% (45)	0	1.45% (9)
Peripheral nerves	0.06% (5)	3.75% (83)	2.41% (33)	5.33% (33)
Complications				
None	90.42% (7370)	77.82% (1723)	83.94% (1150)	73.99% (458)
Thrombosis	0.04% (3)	1.67% (37)	1.09% (15)	2.42% (15)
Embolism	0.05% (4)	1.04% (23)	0.80% (11)	1.45% (9)
Acute respiratory distress syndrome	0.01% (1)	2.21% (49)	0.22% (3)	2.91% (18)
Multiorgan failure	0.01% (1)	2.30% (51)	0.29% (4)	2.91% (18)
Neurological	1.04% (85)	2.98% (66)	2.04% (28)	2.23% (20)
Superficial infection	0.04% (3)	1.26% (28)	0.88% (12)	1.13% (7)
Deep infection	0.09% (7)	3.16% (70)	1.90% (26)	4.68% (29)
Hemorrhage	0.09% (7)	1.67% (37)	1.68% (23)	3.55% (2)
Hematoma	0.17% (14)	1.40% (31)	1.46% (20)	2.58% (16)
Seroma	0.01% (1)	0.63% (14)	0.44% (6)	0.97% (6)
Wound healing disorder	0	0.72% (16)	0.29% (4)	1.62% (10)
Implant loosening	0.02% (2)	1.76% (39)	0.44% (6)	0.97% (6)
Implant failure	0.01% (1)	0.90% (20)	0.36% (5)	0.81% (5)
Secondary fracture dislocation	0.04% (3)	0.86% (19)	0.36% (5)	0.97% (6)
Other	0.65% (53)	8.81% (195)	6.86% (94)	9.21% (57)

## Discussion

In this study, we evaluated the operative and clinical outcomes between patients with pelvic fractures and patients with a combined spine/pelvic injuries in retrospectively collected cohort data from the GPR. We identified a significant association between pelvic fractures and spine injuries. Previous studies have found association between pelvic fractures and other injuries. A radiological study reported that 46% of patients with pelvic fractures had concurrent abdominal injury [31]. The pelvic and abdominal organs most likely to be injured in patients with pelvic fractures are the urogenital tract, major blood vessels, spleen, liver, and kidneys [14, 35, 49]. Meanwhile, extrapelvic injuries mostly to the thorax and head [2, 23, 45]. ﻿Although our study identified a significant relationship between pelvic fractures and spine injuries, it must be noted that this association was predominantly determined by the high frequency of lumbar spine injuries.

Literature review revealed similar satisfactory outcomes after both operative and non-operative management of spinal injuries, thus forming a comparative control group [9, 32, 38, 47]. However, the results are not so encouraging for pelvic fractures [12, 15, 16, 50, 51]. The treatment strategy for pelvic ring or acetabular fractures is decided by the degree of instability or dislocation, as well as the presence of other injuries. Although many pelvic fractures can be treated conservatively, when the fracture pattern involves the posterior pelvic ring and/or there is displacement of the acetabular fracture, ORIF is usually required. In the case that patient’s hemodynamic status allows a surgery and enough surgical experience is available, definitive treatment within the first 24 h after the accident guarantee good clinical and surgical results [11]. New operative techniques are on the rise, such as percutaneous fixation using the 3D fluoroscopic image-based navigation system that produces an intraoperative image comparable to that obtained with postoperative CT, thus allowing the best possible anatomical reduction to be achieved in a single surgical procedure [10]. The pelvic fracture that occurs with a high-energy trauma is often accompanied by spinal cord injury, which may delay osteosynthetic fixation of pelvic fractures.

A delay of more than 3 weeks has been shown to be associated with poor fracture reduction quality and surgical outcome [30].

According to the literature, the mortality rate in patients with pelvic fractures is in the range of 6%-13%, with the lower rates being reported in recent decades [4, 5, 20, 27]. Severe bleeding is the leading cause of death, whether caused by the fracture itself or by other related injuries. Application of standardized trauma management strategies (e.g. ATLS®, MARCH®) in prehospital care and emergency departments can significantly improve outcomes; these strategies involve early aggressive blood and clotting factors transfusion regimens, as well as non-invasive pelvic stabilization (e.g. pelvic binder) [6, 17, 24, 25, 46]. The treatment of pelvic fractures depends on the patient’s hemodynamic status and concomitant diseases. Unstable anterior pelvic ring fractures can be urgently stabilized with an external fixator and the posterior pelvic ring fractures with a pelvic C-clamp.

In the case of uncontrollable bleeding in hemodynamically stable patients, interventional radiological embolization is a viable option, but for hemodynamically unstable patients the gold standard is surgical preperitoneal pelvic packing until the patient’s status allows a revision surgery [36, 39].

A cooperative multidisciplinary approach is necessary to improve outcomes. Protocol-guided bleeding management, a decision-making algorithm, and the involvement of specialized orthopedic pelvic surgeons are essential [40]. Delay of fracture fixation because of concomitant injuries leads to increased morbidity, prolonged immobilization [52], and need for intensive unit care. Effective trauma care can ensure an improved outcome with less inpatient complications and briefer hospital stay, as well as a reduce in clinical resource consumption and costs [53].

In our cohort, patients with pelvic fracture plus spine injury were significantly younger; this correlates with the fact that patients with stable pelvic injury were considerably older (> 65 years of age; isolated pelvic injury 44.8% vs. associated pelvic/spine 8%) and patients with an unstable pelvic injury suffer an association of spine/pelvic injury thinkable of the necessary high-energy required trauma forces. In our cohort, patients with spine plus pelvic injuries had lower Hb levels and blood pressure values at admission. Due to the accompanying spine injuries, these patients were more likely to undergo emergency stabilizations (e.g., external fixation or pelvic C-clamp). Meanwhile, patients with isolated pelvic injury were more likely to receive definitive pelvic fixation. The time until emergency stabilization and the time until definitive pelvic stabilization were both significantly longer in patients with pelvic fracture plus spine injury. Even though, the time until definitive surgical treatment of pelvic fracture was delayed in patients with associated spine injury, the surgery was still performed within the recommended 5–8 days after the accident.

As hypothesized, the outcome was clearly worse when pelvic fracture was associated with spine injury. Patients with combined spine and pelvic injury had longer hospital stay, more blood transfusion within 12 h of admission, more inpatient complications, and higher overall morbidity, mortality, and neurological disability rates.

### Limitations

The main strength of this study is the inclusion of a large sample number of patients from several centers. However, there are limitations. Besides the retrospective nature of the study, one of the main deficiencies is the fact that the German Pelvic Register does not have detailed data related to adjuvant spinal injuries, as it focuses on the treatment of pelvic fractures. To evaluate the effect of different spinal injuries on the quality of care of pelvic fractures, a prospective detailed study will be necessary.

## Conclusion

Delaying definitive surgical treatment of pelvic fractures due to spinal cord injury appears to have a negative impact on the outcome of pelvic fractures, especially on the quality of reduction of acetabular fractures. Severe accompanying injuries increase morbidity and mortality risk thereby prolonging hospital stay. Interdisciplinary consultation and management are necessary to improve outcomes.

## Data Availability

The datasets used and/or analyzed during the current study are available from the corresponding author on reasonable request.

## Abbreviations

AUC: Akademie für unfallchirurgie (Academy of trauma surgery)
AIS: Abbreviated injury score
ATLS: Advanced trauma life support
ANOVA: Analysis of variance
CT: Computer-tomography
GPR: German pelvic registry
Hb: Hemoglobin
ISS: Injury severity score
KIT: Karlsruhe institute of technology)
MARCH®: Algorithm for massive hemorrhage, airway, respiration, circulation, head injury/hypothermia
ORIF: Open reduction and internal fixation
SBP: Systolic blood pressure
